# *Eryngium Billardieri* Induces Apoptosis via *Bax* Gene Expression in Pancreatic Cancer Cells

**DOI:** 10.15171/apb.2018.075

**Published:** 2018-11-29

**Authors:** Neda Roshanravan, Parina Asgharian, Hassan Dariushnejad, Naimeh Mesri Alamdari, Behzad Mansoori, Ali Mohammadi, Shahriar Alipour, Meisam Barati, Abed Ghavami, Vajihe Ghorbanzadeh, Fatemeh Aamazadeh, Alireza Ostadrahimi

**Affiliations:** ^1^Cardiovascular Research Center, Tabriz University of Medical Sciences, Tabriz, Iran.; ^2^Drug Applied Research Center, Tabriz University of Medical Sciences, Tabriz, Iran.; ^3^Department of Pharmacognosy, Tabriz University of Medical Sciences, Tabriz, Iran.; ^4^Department of Medical Biotechnology, Faculty of Medicine, Lorestan University of Medical Sciences, Khorramabad, Iran.; ^5^Students Research Committee, School of Health, Iran University of Medical Science, Tehran, Iran.; ^6^Immunology Research Center, Tabriz University of Medical Sciences, Tabriz, Iran.; ^7^Department of Molecular Medicine, Connective Tissue Disease Research Center, Tabriz University of Medical Sciences, Tabriz,Iran.; ^8^Students Research Committee, Cellular and Molecular Nutrition Department, Shahid Beheshti University of Medical Sciences, Tehran, Iran.; ^9^Nutrition Research Center, School of Nutrition, Tabriz University of Medical Science, Tabriz, Iran.; ^10^Razi Herbal Medicine Research Center, Lorestan University of Medical Sciences, Khorramabad, Iran.

**Keywords:** Adenocarcinoma, Bax, Cyclin D1, Eryngium, Pancreas

## Abstract

***Purpose:*** Pancreatic adenocarcinoma has a high prevalence all over the world. Most of the therapeutic approaches failed as a result of tumor invasion and rapid metastasis. Several natural plants have been shown to have promising therapeutic effects. Thus, the aim of this study was to investigate the cytotoxic activity of Eryngium billardieri against PANC-1 cancer cell lines.

***Methods:*** Dimethylthiazole diphenyltetrazolium bromide assay (MTT assay) and ﬂow cytometry were used to assess the cytotoxicity of E. billardieri extracts against PANC-1 cancer cell lines. Quantitative Polymerase Chain Reaction (qPCR) was conducted to investigate the expression levels of Bcl2- associated X protein (BAX) and cyclin D1.

***Results:*** The results of the MTT assay showed that E. billardieri extracts had cytotoxic effects on PANC- 1 cancer cell lines. Moreover, the ﬁndings from the gene expression confirmed the over expression of Bax, and under expression of cyclin D1 following treatment with dichloromethane (DCM) and n-hexane (n- hex) extracts in cancer cells (P < 0.05). Interestingly, the ﬂow cytometry results showed that DCM and n- hex extracts of E. billardieri induced apoptosis in PANC- 1 cancer cell lines.

***Conclusion:*** The results of this study demonstrated that DCM and n- hex extracts of E. billardieri significantly induce apoptosis by increasing Bax and decreasing cyclin D1 mRNA expression. Therefore, E. billardieri may be regarded as a novel approach for treatment of pancreatic cancer as a result of its promising apoptotic and cytotoxic properties.

## Introduction

Pancreatic adenocarcinoma is one of the most lethal malignant neoplasms across the world, and is associated with the lowest 5-year survival rate.^1^ According to the GLOBOCAN 2012 estimates, pancreatic cancer accounts for more than 331000 deaths per year, making it the third leading cause of cancer death in both sexes together.^2^ The major barrier to positive clinical outcomes for this type of cancer is delayed diagnosis and resistance to existing malignancy therapeutics.^3^

Apoptosis, as a major mechanism which regulates pathways to control cell proliferation and death, is suggested for consideration as one of the targeted therapy strategies for tumor cells.^4^ In recent years, a number of investigations have been carried out that show novel strategies for more successful prevention and therapy of pancreatic cancer. Previous studies demonstrated that *cyclin D1* and *Bax* genes are the most important regulators in controlling the proliferation and apoptosis of cells.^5^

*Cyclin D1* protein plays a crucial role in regulating the progress of the cell during the G1 phase of the cell cycle. The *cyclin D1* gene is intensified in pancreatic carcinomas.^6^ Proapoptotic *Bax* protein can induce apoptosis via the intrinsic signaling pathway (also known as mitochondrial apoptosis).^7,8^ Previous findings suggest that enhanced apoptosis-promoting *Bax* gene expression may have therapeutic application in pancreatic cancer cells.^9,10^

**Figure 1:**
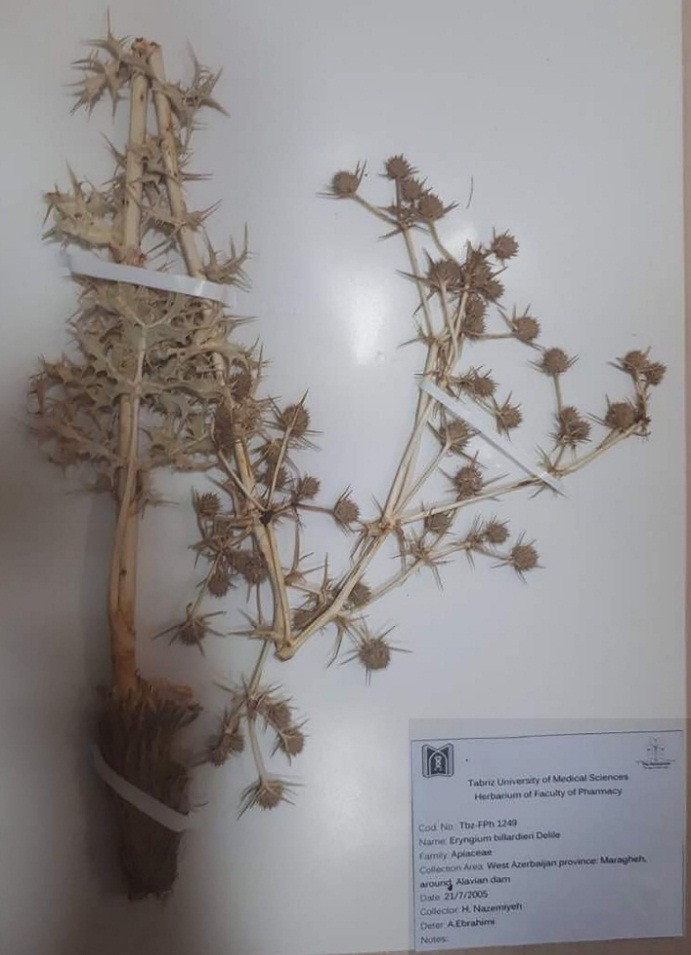
*Eryngium billardieri*.

Pioneering clinical studies have reported that medicinal herbs and their derivative phytocompounds can be considered as useful complementary treatments for cancer.^11,12^*E. billardieri* (Figure 1) belongs to the Umbelliferae family which is used extensively as a medicinal plant worldwide for the treatment of various inflammatory disorders.^13^ In Iranian folk medicine, various parts of this plant are used for a wide range of ailments; such as various inflammatory disorders, rheumatism, sinusitis, wound healing, urinary infections, scorpion bites, goiter etc.^13,14^ Extracts obtained from the root and aerial parts of *E. billardieri* previously showed anti-inflammatory and anti-hyperglycemic effects.^15,16^

The dim prognosis of pancreatic adenocarcinoma is due to a lack of molecular pathways regarding disease development. PANC-1 cell lines (Pancreatic cancer cell lines) are currently used as *in vitro* models to assay pancreatic ductal adenocarcinoma carcinogenesis. The morphological and genetic characteristics of these cell lines are well- known already.^17^ In continuation of our scientific works on the evaluation of the anti-proliferative activity of the Iranian Medicinal plants,^18-25^ this study investigated the cytotoxic activity of n-hex, DCM and methanol (Met) extracts of the aerial parts of *E. billardieri* against PANC-1 cancer cell lines via *Bax* and *cyclin D1* mRNA expression.

## Materials and Methods

### Materials

Methanol, L-glutamine, Penicillin-Streptomycin, MTT, Phosphate-buffered saline (PBS), Trypsin-Ethylenediaminetetraacetic acid (EDTA) solution, and RPMI 1640 were purchased from Sigma (Sigma, St. Louis, MO, USA). Fetal bovine serum (FBS) (HyClone, Logan, UT, USA), Dimethyl sulfoxide (DMSO), Diethyl pyrocarbonate (DEPC) water (Merck, Germany), AnnexinV-FITC/PI apoptosis kit (Invitrogen, USA), SYBR Green PCR master mix (Takara Bio Inc., Tokyo, Japan) were used in this study.

Plant materials were collected from Maragheh- Mountains (Eastern Azerbaijan Province, Iran). Voucher specimens were authenticated by Herbarium of Faculty of Pharmacy, Tabriz University of Medical Sciences. The fresh aerial parts of *E. billardieri* (100 g) were extracted with 1.1 L of n-hex, DCM and MeOH solvents by Soxhlet apparatus, respectively. Then, to reach the highest purity percentage, the extract was filtered by Whatman ﬁlter paper no.40 and was placed in the temperature of 36°C. To yield a dry and concentrated extract, rotary vacuum evaporator was used. Extracts were kept in sterile screw-capped containers and were stored at 6°C until use.

PANC-1 and human embryonic kidney normal cell line (KDR/293) were obtained from the Pasteur Institute, National cell bank, Tehran, Iran. The cells were grown in RPMI 1640 medium (Sigma, St. Louis, MO, USA) which were supplemented with 10% FBS, penicillin G (100 U/ml), and streptomycin (100 ‏µg/ml). Cells were cultured in 25 cm2 culture T- flasks at 37°C in humidified air with 5% CO2.

### MTT assay, gene expression and flow cytometry

**Figure 2:**
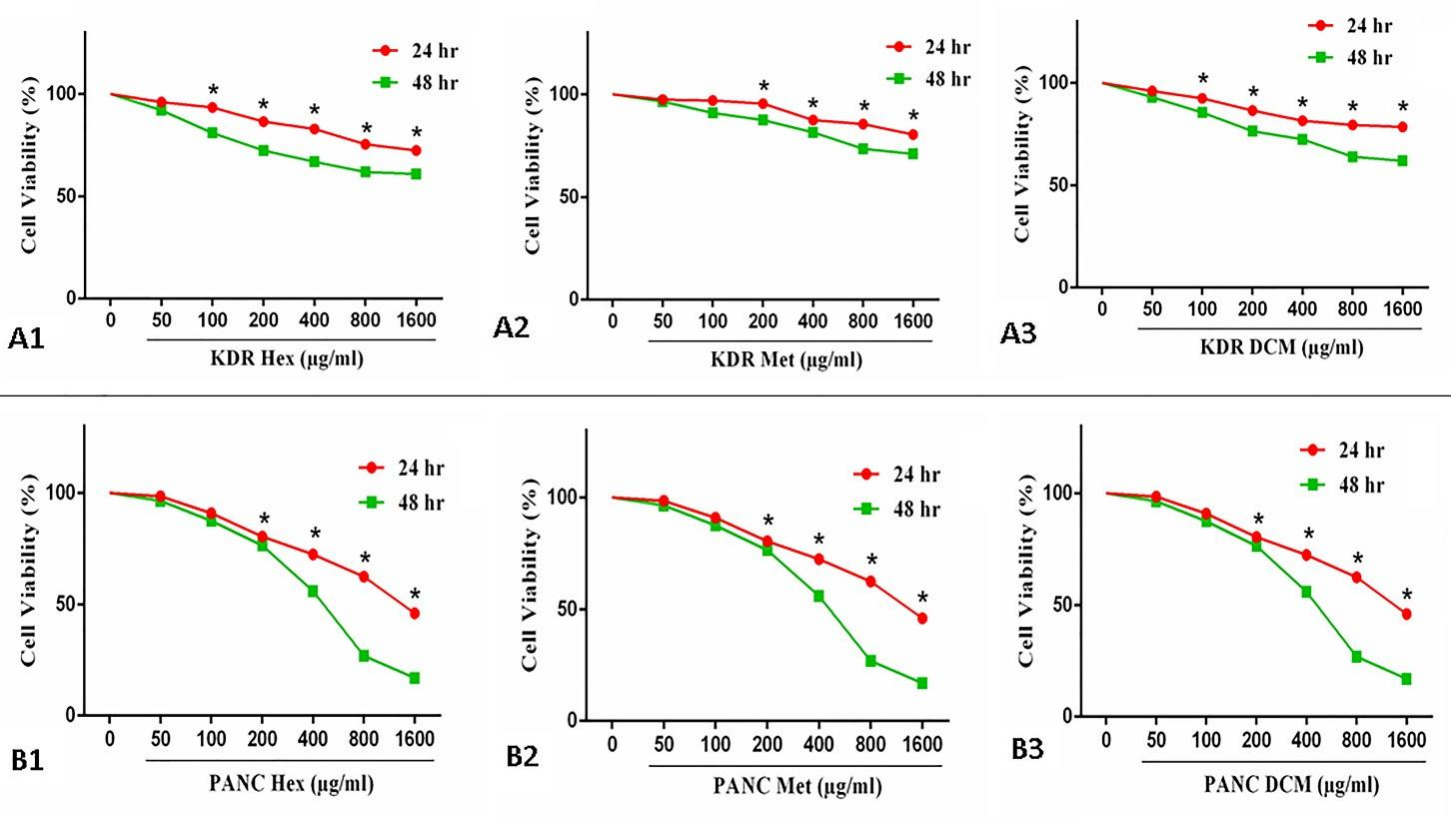
Diagram representing the cytotoxic effect of the *Eryngium billardieri* extract on PANC-1 and KDR/293 cells at 24 & 48 h. A1: KDR Hex, A2: KDR Met, A3: KDR DCM; B1: PANC Hex, PANC Met, PANC DCM.*p < 0.05. Hex: n-hexane extracts; DCM: dichloromethane extracts; Met: methanol extracts.

*In-vitro*, cytotoxicity was assessed by 3-[4, 5-dimethylthiazol-2-yl]-3, 5-diphenyl tetrazolium bromide for treated and untreated PANC- 1 and KDR/293 cell lines. MTT assay is a high accuracy colorimetric method that is widely used to determine cell viability and cell cytotoxicity, particularly in the expansion of new drugs. The cells were seeded in 96-well plates at a density of 15,000 cells per well in their respective RPMI 1640 media. When the cells were approximately more than 80% confluent were left untreated or treated with DCM, MeOH and n-hex extracts of *E. billardieri*. The half maximal inhibitory concentration (IC_50_) was determined by MTT test in the range of 0 to 1600 µg/mL at 24 and 48 hours (Figure 2).

Analyses of *Bax* and cyclin D1 genes expression were all performed after 48 hours of *E. billardieri* treatment by reverse transcription-polymerase chain reaction. Total RNA was extracted from cultured cell lines using TRIzol reagent (Invitrogen, USA) according to the manufacturer's instructions. *β- actin* was considered as an internal control. The relative absorbance ratio at A260/280 and A260/230 on a spectrophotometer (NanoDrop One/Onec, Thermo Scientific) was used to confirm RNA quality and quantity. Total RNA was then converted to cDNA by reverse transcription using Prime Script RT Reagent kit (Takara, RR037Q, Japan) according to manufacturer's recommendations for cDNA synthesis. For qRT-PCR, three replicates of each sample were amplified in a 20 μL reaction mixture containing SYBR Green reaction mix (Takara, Japan) and 0.5 mM of primer and analyzed using real-time PCR (Mic –qPCR, Australia). The amount of the *Bax* and cyclin D1 mRNA were normalized against that of the *β- actin* mRNA and the relative mRNA abundance was analyzed using -2^ΔΔCT^ method.^26^ The primer sequences were designed through the use of PrimerBank and summarized in supplementary appendix (Table 1). PANC- 1 and KDR/293 cells were seeded into six-well culture plates (1.0×106 cells/well) with RPMI 1640 media and incubated at growth condition for 48 h. After that, the cells were washed with pre-warmed culture media and were carefully replaced with a prepared fixative solution (pre-warmed RPMI containing 4% formaldehyde). Cells were treated with 50, 100 and 200 µg/mL of DCM, Met, and n-hex extracts of *E. billardieri*. After treatment time point (48 h), the untreated and treated cells were separated by Trypsin-EDTA, and supernatants were thrown away after centrifugation at 900 rpm for 10 min at 28°C. According to the AnnexinV-FITC/PI apoptosis kit (Invitrogen, USA) instructions, the cell pellets were washed once in PBS, then once in 1X Binding Buffer and were centrifuged and disposed supernatant in each phase. After this stage, the cells were resuspended in 100 µl of 1X binding buffer and were transferred into a new 5 ml tube. Then, 5 µl of FITC-conjugated Annexin V was added to 100 μL of the cell suspension and incubated for 15 min under a dark condition at room temperature. After incubation time, the cells were washed with 1X binding buffer and resuspended in 200 μL of 1X binding buffer again. At the final stage, 5 µl of propidium iodide (PI) staining solution was added to the cells and was analyzed by flow cytometry. Quadrant settings were fixed with untreated controls and copied to dot plots of the treated cells. Data analysis was done using FlowJo (Treestar, Inc., San Carlos, CA). The experiment was repeated triplicate.

**Table 1:** Sequence of Genes primers for qRT-PCR

**Gene**	**Primers**	
**Bax**	Forward	TTCTGACGGCAACTTCAACT
	Reverse	CAGCCCATGATGGTTCTGAT
**Cyclin- D1**	Forward	CCACTCCTACGATACGCTACTA
	Reverse	CCAGCATCTCATAAACAGGTCA
**β-actin**	Forward	GGTGAAGGTGACAGCAGT
	Reverse	TGGGGTGGCTTTTAGGAT

### Statistical analysis

Graph Pad Instat 6 software (GraphPad Instant biostatistics, San Diego, CA, USA) was used to test significant differences between various treatment groups. All data are expressed as means ± SD. Analysis of the experimental data was done by using the one-way ANOVA analysis of variance; following Tukey’s post hoc using SPSS (SPSS Inc. Chicago, IL, USA version 16.0). *P*-values less than 0.05 were considered to be significant.

## Results

### E.billardieri extracts had cytotoxic effects on PANC- 1 cancer cell lines

To determine the cytotoxic effects of *E. billardieri* extracts on PANC-1 and KDR/293 cells MTT assay was performed. Data analysis of cells treated with plant extract at 0- 1600 µg/mL concentrations in 24 & 48 h is illustrated in Figure 2. Moreover, the half-maximal inhibitory concentrations (IC_50_) after treatment by various concentrations of DCM, Met and n-hex extracts of *E. billardieri* were determined and presented in Table 2. As seen in Figure 2, due to the treatment by Met, DCM and n-hex extracts of *E. billardieri* the survival rates of PANC- 1 cells were significantly decreased compared with untreated cell lines (p<0.05) in time and dose dependent manner. However, as shown in Figure 2, the extracts had growth inhibitory effect on the KDR/293 normal cell only in high concentrations which IC_50_ could not be defined from the responses.

**Table 2:** The IC_50_ dose of *E. billardieri* extracts on PANC-1 and KDR/293 cells at 24 & 48 h.

Time	24h		48h	
Cell line	PANC-1	KDR	PANC-1	KDR
n-hex	836.2	-	217.3	-
DCM	1128	-	306.8	-
Met	1329	-	448.5	-

n-hex: n-hexane extracts; DCM: dichloromethane extracts; Met: methanol extracts

### DCM and n-hex extracts of E. billardieri influenced on the Bax and Cyclin- D1 mRNA expression in PANC- 1 cancer cell lines

**Figure 3:**
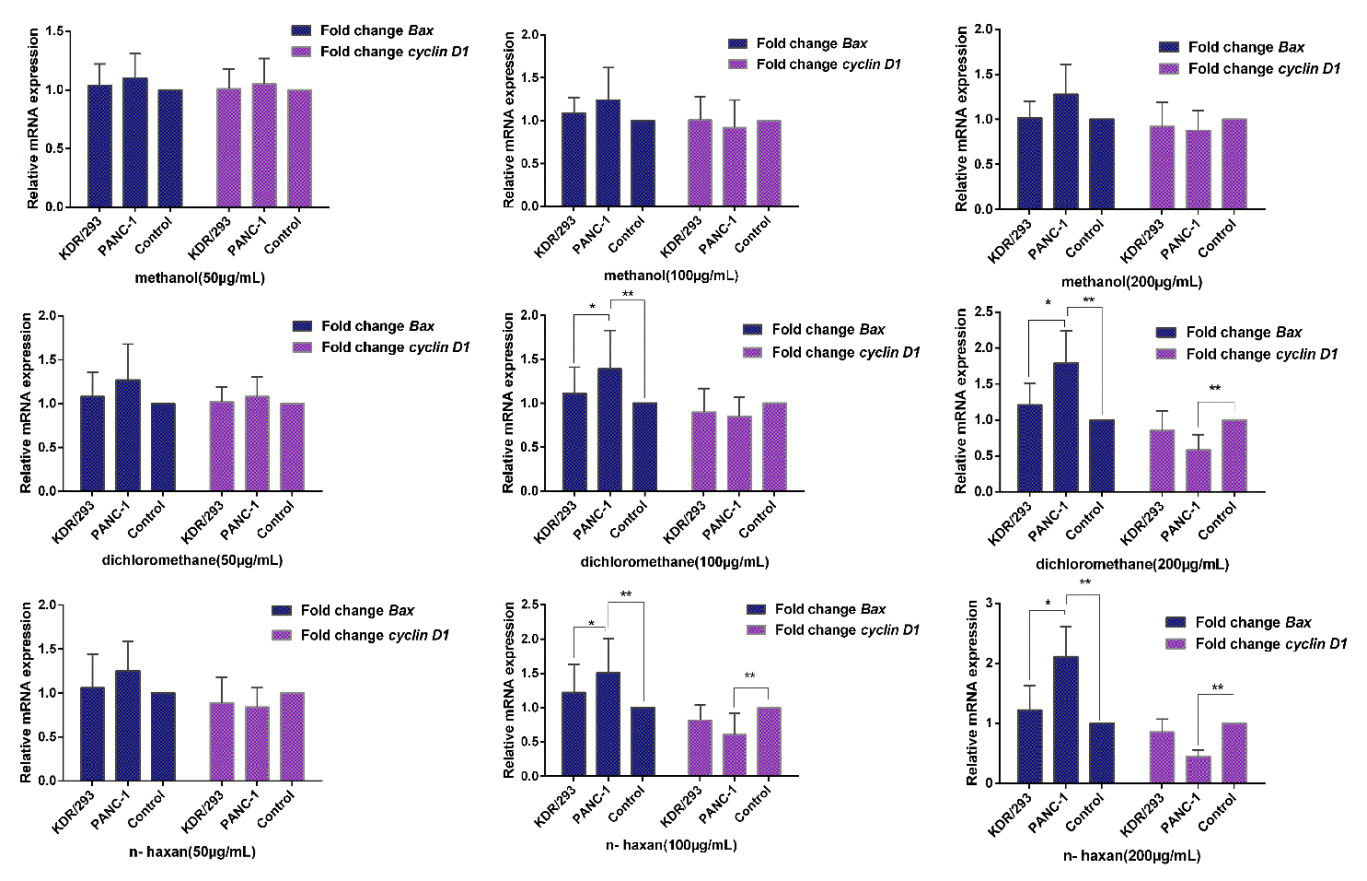
Changes in genes expression in PANC-1 and KDR/293 cell lines treated with the IC_50_ concentration of the *Eryngium billardieri* extracts compared to control group (untreated cell lines). *p< 0.05, **p < 0.05 versus control one (Untreated cells).

We used a QRT-PCR to evaluate the *Bax* and *cyclin D1* expression in PANC- 1 and KDR/293 normal cell lines. According to the results of qRT-PCR (the obtained data are presented in Figure 3), the expression levels of *Bax* were signiﬁcantly increased in PANC-1 cells after treatment with DCM and n-hex extracts with 100 and 200 µg/ mL concentration compared to untreated control cells and KDR/293 (p<0.05). Interestingly, the expression level of *cyclin D1* mRNA decreased after treatment with DCM and n-hex extracts in the treated cancer cells compared to untreated control cells (p<0.05). Furthermore, there were not any significant differences in the expression levels of investigated genes subsequent to exposure KDR/293 normal cells (p>0.05). Unexpectedly, we did not see any difference in the expression levels of *Bax* and *cyclin D1* genes subsequent to exposure cell lines with Met extract of *E. billardieri* (p>0.05). Upon to the results of QRT-PCR, it is obvious that none of the extracts at the concentration of 50µg/ mL had effects on genes expression in PANC-1 and KDR/293 cell lines (p>0.05).

### DCM and n-hex extracts of E. billardieri induced apoptosis in PANC- 1 cancer cell lines

**Figure 4:**
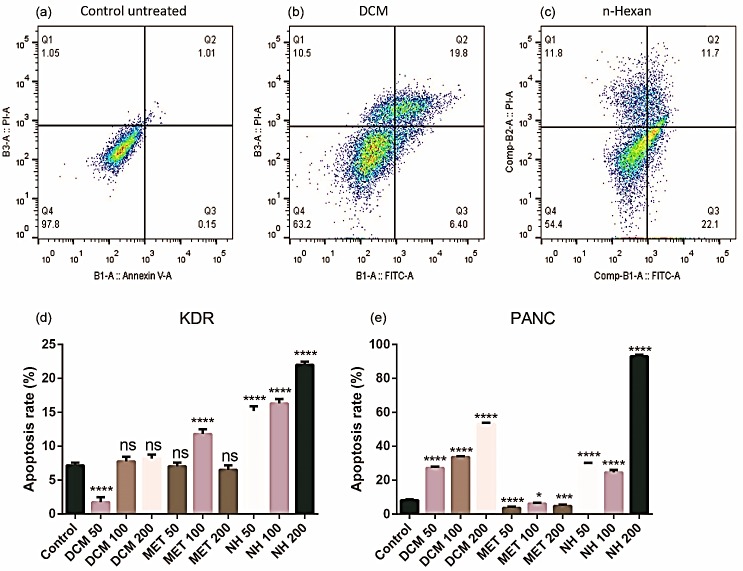
Representative FACS plots of treated/untreated PANC-1 & KDR/293 cells. Cells were incubated with FITC-Annexin V in combination with PI to detect apoptosis and necrosis and classified as necrotic cells (the upper left quadrant (Q1); Annexin −/PI +), late apoptotic cells (the upper right quadrant (Q2); Annexin +/PI +), early apoptotic cells (the lower right quadrant (Q3); Annexin +/PI −) or intact cells (the lower left quadrant (Q4); Annexin −/PI −). (a): control untreated KDR cells, (b): DCM 50 µg/mL on PANC-1 cells, (c): n- hexan 50 µg/mL on PANC-1 cells. (d): Apoptosis rate in KDR cells. (e): Apoptosis rate in PANC-1 cells. *<0.05, ***<0.01, ****<0.001. NH: n-hexane; Met: methanol; DCM: dichloromethane.

Figures 4 showed the representative FACS plots. The ﬂow cytometry results represented that the untreated control KDR/293 and PANC-1 cells that primarily incubated by Annexin V and PI were approximately viable and no apoptosis and no necrosis occurred (97.8% & 85.2% respectively: Annexin V-/ PI-). Meanwhile, treatment with DCM and n-hex extracts in the highest determined dose (200 *µg/ mL)* for 48 h increased the percentage of PANC-1 cells in late (Annexin V+/PI+) apoptosis compared to the untreated control and normal KDR/293 cells. Our results revealed that Met extract of *E. billardieri* couldn’t be able to trigger apoptosis pathway in PANC-1 and KDR/293 cell lines (Annexin V-/PI-). However, DCM and n-hex extracts of* E. billardieri* might initiate apoptosis in KDR/293 cells at slow rate with highest determined dose (200 *µg/ mL)*. In addition, after treatment with DCM and n-hex extracts of *E. billardieri* in three doses four groups including viable cells (Annexin V and PI negative), necrosis (Annexin V negative and PI positive), cells undergoing early apoptosis (Annexin V positive and PI negative), and cells in the late stage of apoptosis (Annexin V and PI positive) appeared. As a result, we can conclude that DCM and n-hex extracts of *E. billardieri* can induce a high level of apoptosis in treated cells (p<0.05).

## Discussion

Pancreatic adenocarcinoma is the third leading cause of cancer-related death. Its highly lethal rate can be attributed to its poor prognosis and despite the advances in surgical intervention and chemotherapy; little effect has been made on the mortality rate of this disease. There is a serious need for complementary therapies with better efficacy. Previous *in vitro* studies about medicinal plants revealed that different species of *Eryngium* have demonstrated biological activities including cytotoxic, apoptotic, antimicrobial and anti-inflammatory.^27^ The focus of the present work was to investigate the molecular pathways including cell cycle arrest and apoptosis of *E. billardieri* and its antitumor cytotoxic effect.

In this study, it was found that DCM and n-hex extracts of *E. billardieri* could induce a high level of apoptosis in PANC-1 treated cells and kept the ratio of necrosis negligible. Moreover, these extracts increased the expression level of *Bax* mRNA in PANC-1 cell lines whereas the *cyclin D1* expression was decreased in the treated cancer cells. As it was expected, the elevated expression of *Bax* mRNA was in line with apoptosis in PANC-1 treated cells.

Cell cycle progression is regulated at both the G to S and G to M transition states as a result of the activation and inactivation of specific protein kinases family and their regulatory subunits including cyclins and catalytic subunits which are termed cyclin-dependent kinases (Cdks). Till now, 11 members of the cyclins have been identified including cyclins A, B1±2, C, D1±3, E, F, G and H. The *cyclin D1* plays a vital role in orchestrating cell progression through the G1 phase of the cell cycle and determines whether a cell will progress towards mitogenesis. Several observational studies have suggested that *cyclin D1* plays a key role in promoting the growth of certain human malignancies.^6,28^ By considering various findings, nowadays it has been established that deregulation of *cyclin D1* expression has contributed to the loss of cell cycle control and enhance tumorigenesis.^29^ A correlation has previously been pointed out between increased *cyclin D1* and cancer formation.^30,31^ Based on the results of this study, the DCM and n-hex extracts of *E. billardieri* reduced *cyclin D1*expression in treated cancer cells with no effect on the normal cells.

We also confirmed that both *E. billardieri* extracts can stimulate *Bax* mRNA expression in PANC-1 cancer cells. *Bax* as a pro-apoptotic protein can trigger apoptosis by increasing the opening of the mitochondrial voltage-dependent anion channels, which induce the loss in its membrane potential.^32^ Our results suggest that the apoptotic mechanisms of n-hex extracts of *E. billardieri* in PANC-1 cells include the down regulation of *cyclin D1* expression and the up regulation of *Bax* expression in a dose-dependent manner.

Our results replicate the ﬁndings of Esmaeili et al.^14^ who indicated the cytotoxic potential effects of *E. billardieri* on MCF-7, A549, HepG-2 and HT-29 cell lines. The main components of *Eryngium* species such as essential oils, sterols, saponin, and sanicula saponin are responsible for its cytotoxic activities. These compounds have shown potent and highly selective inhibition against PANC-1, HL-60, A549, PC-3, and MRC-5 tumor cell lines with almost no cytotoxicity against normal human cells.^33,34^

The hexane extract of various species of *Eryngium* exhibited anti-inflammatory and antioxidant activities in animal models.^35^ It has been mentioned that these effects may be due to a decrease in nitric oxide, inflammatory cytokines and TNF-α synthesis.^36^ In line with several investigations which attempted to find bioactive natural anticancer compounds, that induce apoptosis in cancer cells without cytotoxic effects against normal human cells, we indicated that the cytotoxic dose of DCM and n-hex extracts of *E. billardieri* did not cause a significant change in the survival rate of KDR/293 normal cells. The results of this study indicated that nonpolar extracts of *E. billardieri* were generally more effective than the methanolic ones. Similarly, Roumy et al^37^ showed the high cytotoxic/antiplasmodial effects of the DCM and n-hex extracts of some Amazonian plants. Therefore, the effective inhibition of PANC-1 cancer cells *in vitro* suggests that DCM and n-hex extracts of *E. billardieri* may be potentially promising anticancer agents for the effective treatment of pancreatic cancer cells. In this study, there are some limitations: 1) the examination of anticancer effects on PANC-1 cancer cells results to its effect to be unknown on other pancreatic cancer cell lines. 2) Lack of isolation and investigation of *E. billardieri* constituents.

## Conclusion

In conclusion, the results of the current study are the first to indicate that DCM and n-hex extracts of *E. billardieri* significantly induce apoptosis by increasing *Bax* and decreasing *cyclin D1* mRNA expression. However, more molecular studies for investigating the probable apoptotic pathways of the cytotoxic activity of *E. billardieri* should be conducted to achieve a definite conclusion.

## Acknowledgments

The authors would like to thank the Nutrition Research Center of Tabriz University of Medical Sciences.

This study was funded by Tabriz University of Medical Sciences (grant number: IR.TBZMED.5.553473). The financial supporter of the study had no role in study design, data collection, data analysis, data interpretation, or writing the report. The corresponding author was well informed of all the data in the study and had final responsibility for the decision to submit for publication.

## Ethical Issues

This project has met the principles of the Ethics Committee of Tabriz University of Medical Sciences (Ethical code: IR.TBZMED. REC. 1396. 618).

## Conflict of Interest

The authors declare that they have no conflict of interest.

## Abbreviations

Dimethylthiazole diphenyltetrazolium bromide assay: MTT assay; Quantitative Polymerase Chain Reaction: qPCR; Bcl2- associated X protein: BAX; Phosphate-buffered saline: PBS; Ethylenediaminetetraacetic acid: EDTA; Fetal bovine serum: FBS; Dimethyl sulfoxide: DMSO; Diethyl pyrocarbonate: DEPC; half maximal inhibitory concentration: IC50; Cyclin-dependent kinases: Cdks
